# Exploring medical and veterinary student perceptions and communication preferences related to antimicrobial resistance in Ontario, Canada using qualitative methods

**DOI:** 10.1186/s12889-023-15193-x

**Published:** 2023-03-13

**Authors:** Courtney A. Primeau, Jennifer E. McWhirter, Carolee Carson, Scott A. McEwen, E. Jane Parmley

**Affiliations:** 1 Department of Population Medicine, Ontario Veterinary College, University of Guelph, N1G 2W1 Guelph, ON Canada; 2 Centre for Food-borne, Environmental and Zoonotic Infectious Disease, Public Health Agency of Canada, N1H 7M7 Guelph, ON Canada

**Keywords:** Antimicrobial resistance, Qualitative study, Canada, One health, Focus groups, Medical students, Veterinary students

## Abstract

**Background:**

Antimicrobial resistance (AMR) threatens our ability to treat and prevent infectious diseases worldwide. A significant driver of AMR is antimicrobial use (AMU) in human and veterinary medicine. Therefore, education and awareness of AMR among antimicrobial prescribers is critical. Human and animal health professionals play important roles in the AMR issue, both as contributors to the emergence of AMR, and as potential developers and implementers of effective solutions. Studies have shown that engaging stakeholders prior to developing communication materials can increase relevance, awareness, and dissemination of research findings and communication materials. As future antimicrobial prescribers, medical and veterinary students’ perspectives on AMR, as well as their preferences for future communication materials, are important. The first objective of this study was to explore medical and veterinary student perceptions and understanding of factors associated with emergence and spread of AMR. The second objective was to identify key messages, knowledge translation and transfer (KTT) methods, and dissemination strategies for communication of AMR information to these groups.

**Methods:**

Beginning in November 2018, focus groups were conducted with medical and veterinary students in Ontario, Canada. A semi-structured format, using standardized open-ended questions and follow-up probing questions was followed. Thematic analysis was used to identify and analyze patterns within the data.

**Results:**

Analyses showed that students believed AMR to be an important global issue and identified AMU in food-producing animals and human medicine as the main drivers of AMR. Students also highlighted the need to address society’s reliance on antimicrobials and the importance of collaboration between different sectors to effectively reduce the emergence and transmission of AMR. When assessing different communication materials, students felt that although infographics provide easily digestible information, other KTT materials such as fact sheets are better at providing more information without overwhelming the target audiences (e.g., professional or general public).

**Conclusion:**

Overall, the study participants felt that AMR is an important issue and emphasized the need to develop different KTT tools for different audiences. This research will help inform the development of future communication materials, and support development of AMR-KTT tools tailored to the needs of different student and professional groups.

**Supplementary Information:**

The online version contains supplementary material available at 10.1186/s12889-023-15193-x.

## Background

Antimicrobial resistance (AMR) has emerged as a significant threat to public health, with adverse health consequences including increased frequency, duration, and severity of infection [1–3]. In 2018, approximately 26% of select reported human infections in Canada were resistant to first-line antimicrobials, resulting in an estimated 5,400 lives lost per year as a direct result of AMR [4]. By 2050, the prevalence of resistant human infections is expected to increase to 40% in Canada, which could result in 13,700 lives lost each year as a direct result of AMR [4]. AMR is a highly complex issue with the potential to impact several societal sectors, including human and animal health, the economy, the environment, and animal and plant production [5–8]. Due to the complex nature of AMR and its multiple impacts on society, animals, and the environment, a multi-pronged One Health approach that encourages multi-sectoral collaboration is necessary to ensure that antimicrobials remain effective for treatment of infectious diseases [8, 9].

A significant contributor to the emergence and persistence of AMR is antimicrobial use (AMU) in human and veterinary medicine [2, 5, 10, 11]. Professional oversight can help reduce unnecessary use; therefore, antimicrobial prescribers, including both physicians and veterinarians, are important stakeholders to engage in discussion about actions and approaches to reduce AMR. The complexity of AMR poses unique intervention challenges, such as the need to address multiple behaviours and beliefs among different antimicrobial prescribers, dispensers and consumers. Because of the different target groups and behaviours that need to be addressed, it is necessary to engage in audience segmentation, where researchers or policy-makers consider distinct target audiences to develop a comprehensive understanding of the issue within each audience and identify communication methods that are appropriate and accessible for each group. Groups may differ in their attitudes, beliefs, and barriers to change, but all perspectives are relevant to the development of comprehensive strategies to address AMR.

Knowledge translation and communication initiatives in relation to AMR have primarily focused on improving the use of antimicrobials among the general public and physicians [12–14]. These studies found that educational campaigns targeting the public and physicians simultaneously appeared to reduce AMU. For example, an educational program using posters, radio broadcasts, community presentations, and other communication products (e.g., school educational materials, physician treatment guides) targeted at healthcare professionals and the community resulted in an increase in knowledge of appropriate AMU, and a simultaneous reduction in the rate of methicillin-resistant *Staphylococcus aureus* infections [12–14]. Furthermore, a previous study in the United Kingdom (UK) used a survey to assess the knowledge, perceptions, and practices related to AMU and AMR in human and animal health students [15]. Although the study was exploratory and based on a small sample size, the results suggested that both types of students desired more information on the links between the health of humans, animals, and the environment [15]. We are not aware of any previous studies that have explored in-depth medical or veterinary students’ understanding of the wider dimensions (i.e., non-clinical aspects) and variety of drivers contributing to the emergence of AMR.

The need for qualitative research to address AMR has been recognized, as qualitative methodologies can provide a more detailed and broad understanding of the various drivers of AMR and help develop effective interventions [16, 17]. This study therefore used focus groups with medical and veterinary students in Ontario, Canada. The overall objectives of this study were to: (1) explore medical and veterinary student perceptions of AMR and their understanding of the factors associated with AMR emergence and spread, (2) identify relevant content, including key messages, for future AMR communication products for these student groups, (3) identify the most preferred format of knowledge translation and transfer (KTT) communication products for each student group, and (4) identify preferences and strategies for delivering future communication products to each student group.

## Methods

### Study design

The study consisted of four focus groups conducted between November 2018 and April 2019. The study protocol was approved by the University of Guelph Research Ethics Board (REB #18-05-13) and the Public Health Agency of Canada Research Ethics Board (REB #2018-0009) for compliance with guidelines for research involving human participants, and all methods were performed in accordance with these guidelines and regulations. The relevant ethics committees at each participating university also reviewed and approved the study protocol.

### Study participants

Students registered in any year of the Doctor of Veterinary Medicine (DVM) program at the Ontario Veterinary College, the Doctor of Medicine (MD) program at one of six Ontario universities (Queen’s University, University of Toronto, McMaster University, University of Ottawa, University of Western Ontario, Northern Ontario School of Medicine), or the Doctor of Pharmacy program at one of two Ontario universities (University of Toronto, University of Waterloo) who spoke fluent English were eligible for inclusion in this study. Pharmacy students were included in this study given their future role in dispensing antimicrobials. A recruitment email was distributed to all students enrolled at the universities that agreed to disseminate this email. The recruitment email included an information letter and consent form that highlighted the purpose and specific objectives of the study. Written informed consent was obtained from each focus group participant prior to the start of the discussions and each participant was provided with a $25 gift card and pizza and refreshments at the focus group for participating in the study.

### Focus group structure

Focus groups were conducted on the campuses of the Ontario universities participating in the study and were 1.5 to 2 h in length. The discussions were facilitated with the assistance of a note-taker. The focus groups were conducted in English, and a semi-structured format using a series of standardized open-ended questions was followed (Additional file 1). The focus group guide highlighted four main themes: perceptions of AMR, content of future communication products, format of future communication products, and transmission of future communication products. Examples of questions from the focus group guide can be found in Table 1.

**Table 1:** Ontario medical and veterinary students focus group topic guide

Topic	Areas explored
Perceptions of antimicrobial resistance (AMR)	• General knowledge of AMR • The role of their respective health profession in the issue of AMR
Content of future communication materials	• Key messages that future physicians/veterinarians/pharmacists need to know about AMR • Key messages that future patients/clients need to know about AMR
Format of future communication materials	• Assessment of different types of communication materials, such as infographic, short summary, fact sheet, and a long report
Transmission/delivery of future communication materials	• Most effective ways of receiving and accessing information about AMR as future health professionals

After completing the introductory questions in the focus group guide, a slide deck about AMR was presented to participants (Additional file 2). The slides described AMR and its importance; they also highlighted why antimicrobials are important for humans, animals, and plants. These slides were developed by the research team to provide all participants with a baseline introduction to AMR, as we expected there to be differing levels of awareness and understanding of the issue among participants. Participants were also shown example communication products that were obtained online, including infographics, short reports, fact sheets, and long reports (Additional file 3). All focus groups were recorded with consent using a digital audio recorder.

### Pre- and post-focus group survey

Prior to the focus group discussions and presentation of the AMR slide deck, participants were asked to complete an anonymous survey (Additional file 4). The survey assessed how participants would rank the issue of AMR in terms of importance relative to other global issues (e.g., climate change, terrorism, food security, chronic diseases) and assessed their general knowledge of the drivers and impacts of AMR. The purpose of the survey was to inspire thinking about the topic of AMR and to provide the research team with some context around the ideas that emerged, as participants were expected to vary in their baseline knowledge of the topic. The survey was re-administered at the end of the focus group to assess whether participant beliefs, attitudes, and understanding had changed.

### Data analysis

All identifying information provided by the participants was removed upon transcription of the audio recordings. The principles of inductive thematic analysis were used to identify, analyze, and report patterns within the data [18]. Briefly, the focus group data were transcribed verbatim and read several times by the primary researcher. A codebook including codes and accompanying definitions was developed and used to code the transcripts. A subset of the focus group transcripts were coded by two additional reviewers to ensure the codebook was valid and reliable for coding the data, and to refine the codebook when necessary [19]. Discrepancies in coding were discussed until consensus was reached. Codes were then collated into potential sub-themes and larger themes to develop a thematic map of the data. Additionally, descriptive statistics using Microsoft Excel 2016 were used to analyze the results of the pre- and post-focus group surveys.

## Results

### Study participants

Out of the six Ontario medical schools, four medical schools agreed to disseminate the recruitment email to their students. Out of the two Ontario pharmacy schools, one pharmacy school agreed to disseminate the recruitment email to their students. A total of four focus groups were conducted: one with medical students, and three with veterinary students., We attempted to recruit students at one Ontario pharmacy school but were unable to conduct any focus groups with this population due to a lack of response to recruitment emails.

A total of 18 students participated in the focus groups: 13 veterinary students (three focus groups) and five medical students (one focus group). Of the veterinary students, eight were in their first year of study, three were in second year, one student in third year, and one in fourth year. Four medical students were in their first year of study, and one student was in second year.

### Pre- and post-focus group survey

Student participants were surveyed about their general knowledge of AMR, including the drivers and impacts of the issue (Table 2). When asked about the impacts of AMR in the pre-focus group survey, all students indicated that AMR can affect human and animal health, and the majority of students (89%) indicated that AMR can impact the environment. When asked about the drivers of AMR, all students indicated that human antimicrobial use (AMU) in the community, and AMU in food-producing animals were contributors. Antimicrobial use in hospitalized people and in companion animals were identified as contributors to AMR by 94% of students. Furthermore, when asked if they were familiar with the terms “antibiotic stewardship” or “antimicrobial stewardship”, over one-third (39%) of students indicated that were unsure or had not heard of either term. A greater proportion of veterinary students were familiar with the terms compared to medical students (69% vs. 40%, respectively).

**Table 2:** Results of the pre-focus group survey on antimicrobial resistance knowledge administered to study participants

**Question**: Antimicrobial resistance can impact which of the following (select all that apply):			
	**All students (n = 18)**	**Veterinary students (n = 13)**	**Medical students (n = 5)**
The environment	89%	92%	80%
Human health	100%	100%	100%
Animal health	100%	100%	100%
Unsure	0%	0%	0%
**Question**: In your opinion, which of the following contributes to the issue of antimicrobial resistance? Select all that apply.			
	**All students (n = 18)**	**Veterinary students (n = 13)**	**Medical students (n = 5)**
Antimicrobial use in hospitals	94%	92%	100%
Antimicrobial use in the environment	89%	92%	80%
Antimicrobial use in the community	100%	100%	100%
Antimicrobial use in companion animals (e.g. dogs, cats)	94%	100%	80%
Antimicrobial use in food-producing animals (e.g. cattle, pigs, chicken)	100%	100%	100%
Other (please specify)	0%	0%	0%
**Question**: Have you ever heard of the term “antibiotic stewardship” or “antimicrobial stewardship”?			
	**All students (n = 18)**	**Veterinary students (n = 13)**	**Medical students (n = 5)**
Yes	61%	69%	40%
No	28%	31%	20%
Unsure	11%	0%	40%

Participants were also asked to rank AMR in terms of its importance relative to other global challenges on a scale of 1 to 5, with a score of 5 indicating an extremely important issue (Table 3). Prior to the focus group discussions, the ranking of AMR (mean score of 3.3) was higher than other global challenges apart from climate change (mean score of 3.8); this remained the same in the post survey. The veterinary student participants had generally stable rankings pre- and post-focus group. In contrast, the medical student participants ranked AMR as less important than most other global challenges pre-focus group, but increased the ranking of the importance of AMR post-focus group.

**Table 3:** Ranking of the perceived importance of antimicrobial resistance relative to other global challenges amongst study participants*

Global challenge	All students (n = 18)		Veterinary students (n = 13)		Medical students (n = 5)	
	*Pre-focus group*	*Post-focus group*	*Pre-focus group*	*Post-focus group*	*Pre-focus group*	*Post-focus group*
Antimicrobial Resistance	3.3	3.3	3.5	3.5	2.8	3.4
Climate Change	3.8	3.8	4.0	4.1	3.2	3.0
Terrorism	3.0	2.8	2.6	2.5	4.0	2.8
Food Security	2.6	2.6	2.6	2.5	2.4	2.8
Chronic Diseases	2.4	2.6	2.3	2.3	2.6	3.2

*Using a scale of 1 to 5, where a ranking of 1 = not important, 5 = extremely important

### Thematic analysis

**Fig. 1:**
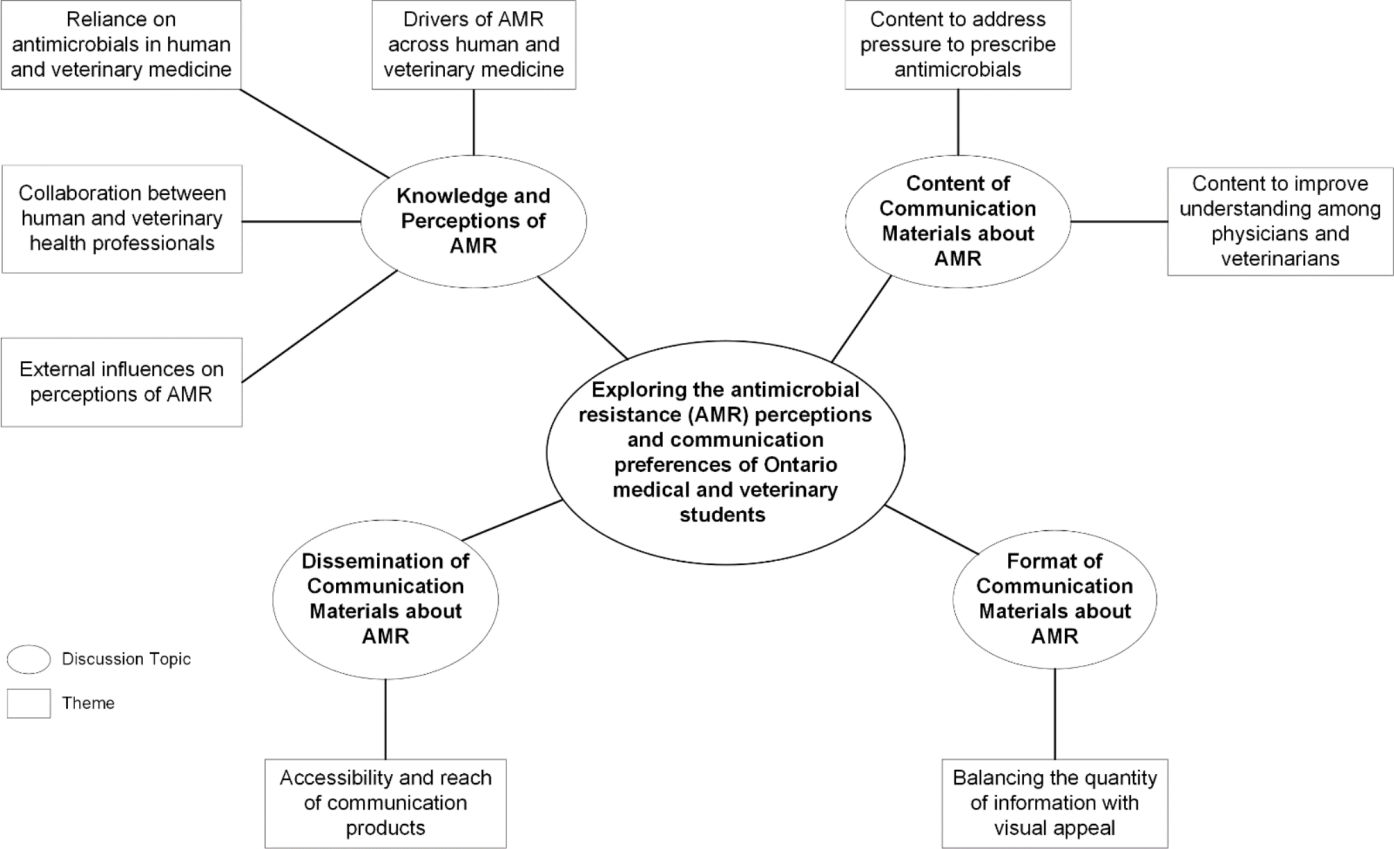
Themes emerging from the focus groups about the perceptions of antimicrobial resistance, and communication preferences

The results of the thematic analysis of the focus group transcripts were organized into four broad topics based on the focus group guide, including knowledge and perceptions of AMR, content of AMR communication products, format of AMR communication products, and transmission of AMR communication products (Fig. 1). Several themes emerged from the analysis of the focus group transcripts, and will be discussed in the following sections.

### Knowledge and perceptions of AMR

#### Theme 1: Reliance on antimicrobials in human and veterinary medicine

The medical and veterinary students participating in the focus groups discussed the importance of AMR in depth, and often commented on the reliance on antimicrobials in their respective fields. Veterinary students felt that this reliance was a significant issue with many implications for the practice of veterinary medicine in the future. These participants described the potential impacts of AMR in a world without effective antimicrobials, explaining that it poses a threat to the spread and treatment of infectious diseases. The veterinary students also highlighted that AMR presents significant animal welfare issues, with one participant stating that “We’re going to get to a point where we aren’t going to be able to treat anything, and you are just going to have to see these animals suffer.” Medical students also described the importance of AMR and the need to preserve the effectiveness of antimicrobials. As one medical student stated, “Antibiotics are the biggest medical revolution we’ve ever had. They vastly improved people’s quality of life, length of life. […] We want them to continue working.”

#### Theme 2: drivers of AMR across human and veterinary medicine

Participants in all focus groups highlighted AMU as an important driver of AMR. Across the veterinary focus groups, most participants focused their AMU discussions on the overuse of antimicrobials in both production animals and human medicine and felt that overuse in these sectors was the largest contributor to the emergence of AMR. Many veterinary student participants felt that the media and society placed an undue burden on veterinarians and food producers in terms of responsibility for AMU and AMR and felt that AMU in human medicine was just as important, but often overlooked. In contrast, one veterinary student felt that there was greater awareness of AMU in human medicine compared to veterinary medicine, explaining that “I’ve always heard more about antibiotic use in humans, and you don’t hear much for veterinary usage. In the small animal sector, you don’t really think of it as much of an issue […] All streams of veterinary medicine need to be involved in trying to reduce use, not just the food production sector.”

Interestingly, participants in the medical student focus group emphasized the role of AMU in production animals relative to human AMU in the emergence of AMR, and this was raised several times during the discussion, despite these participants stating they lacked a thorough understanding of AMR and its links to the agricultural sector. One participant stated that “…I know that you see a lot more routine use. I think that kind of caused resistance. But I’m not really sure about but I can talk based on what I’ve seen in the media. It’s just always kind of used in regular cow rearing and stuff.” Another medical student participant followed up on this comment, explaining that “I have this notion that it was about like overcrowding and farms and stuff and in order to get more animals into smaller spaces to make meat cheaper, they used a lot of prophylactic antibiotics to just stop it from spreading…”.

The influence of patient pressure on physicians was reported by medical students to be an important driver of antimicrobial prescribing practices, and therefore something to consider when addressing AMR. Most of the medical student participants described having experienced this firsthand when completing placements at family health clinics, and also discussed how they have observed physicians “…giving in to patient demands” to find a “quick and easy solution, which you need because you don’t have much time to spend with each patient that comes into the clinic”. One medical student participant described their experience in a clinic, explaining that “…When I was doing a placement and I went to see two children with some kind of chest infection, we didn’t know if it was viral or bacterial. The parents kept sitting there, suggesting antibiotics. We kept trying to explain it to them, kept trying to explain and eventually the physician was just so frustrated, so he gave them the antibiotic so they would just leave… we aren’t taught what to do when patients are demanding a certain course of action. These are hard scenarios and sometimes we do get told, sometimes you just kind of gotta give in.”

Similarly, the idea of client pressure to prescribe emerged across most of the focus groups conducted with veterinary students. They described the barriers that veterinarians face when it comes to diagnostic testing and attempting to identify a causative agent for a sick animal. For example, participants felt that owners of companion animals may face financial constraints and be unwilling or unable to pay for proper diagnostic tests for their pets. As a result, owners will “come in and ask for an antibiotic, hoping that it will help their pet, rather than pay to get blood work or other tests done to find out what is really going on”. Veterinary students felt that determining the cause of illness in animals is more difficult than in humans, because “if you need to do more diagnostic tests to be sure you are prescribing correctly, those are added costs that the clients may not want to do. You get it all the time where someone will call and say their cat has a UTI [urinary tract infection] again, and I just want to refill the antibiotic again.”

#### Theme 3: external influences on perceptions of AMR

The role of external influences (e.g., family members, peers, the media, industry) on the understanding and perceptions of AMR was discussed in depth across all focus groups. Both veterinary and medical students felt that the media had a significant impact on understanding and perceptions of AMR, and they believed this to be true for both themselves and members of the general public. In the veterinary student focus groups, students explained that media coverage can raise general public awareness of an issue, with one student explaining that “The messages that are communicated through the media tend to resonate with people, whether those messages are correct or incorrect…it’s also such a good tool to reach a wide variety of audiences.” Additionally, the amount of media coverage attributed to global issues was discussed by the medical students, and they felt that the amount of media coverage an issue receives can significantly influence perception of the importance of the issue. Students in this focus group generally felt that AMR received less media coverage compared to other global issues, with one student explaining that “I don’t think that AMR gets equal coverage in the media, so people don’t think of it or maybe aren’t as aware of the issue.” Another medical student felt that physicians should be involved in the media coverage of AMR, as people tend to respect the opinions of physicians, explaining that “…there definitely is a role for medical professionals to speak out and say more in the public realm or in the media or whatever that we recognize that this is an issue.”

Additionally, the role of family members and peers in shaping perceptions of AMR was mentioned by medical and veterinary student participants. Students felt that people are very likely to be influenced by those around them, and this may impact their understanding of AMR and increase uncertainty and misconceptions of the drivers of the issue. One medical student reflected on her own upbringing on a farm, and her skepticism of some of the information being conveyed by her family members, explaining that “Like I grew up on a farm and my whole entire family are farmers and I remember one of my relatives telling me at one point, which I don’t know if it’s true or not, that because the antibiotics that we use in animal feed and agriculture are different than the ones prescribed for human illnesses, it is not supposed to interfere with their resistance build up for the types of antibiotics used in hospitals. But I’m a little bit skeptical that that’s exactly true.” The role of medical professionals in influencing medical and veterinary student perceptions was also discussed, with students noting that the professionals responsible for training have their own thoughts and opinions on how best to treat patients, and this can sometimes influence medical or veterinary student perceptions of AMU. Both student groups mentioned that in many cases, prescribing antimicrobials without proper diagnostic tests is a common occurrence. As one veterinary student explained, “I’ve trained under vets who tell clients “we are pretty sure it’s a UTI [urinary tract infection], so here you go, finish the pills”. This sentiment was shared by a medical student participant, stating that “One time when I was in the clinic, people coming in with sore throats and colds and whatever, it seemed very routine that the doctor I was shadowing wouldn’t really think twice about just prescribing antibiotics and saying we would just try it and see what happens. In my head I was thinking, are they even considering this or is it just routine for them? Might be bacterial, might as well prescribe them antibiotics.”

#### Theme 4: collaboration between human and Veterinary Health Professionals

Across all focus groups, the idea of AMR being a shared responsibility among human and veterinary medicine emerged and was discussed in depth. Medical and veterinary students alike felt that both sectors played important roles in AMR, and collaboration between the two fields is critical to effectively address AMR. Additionally, students indicated that because AMU is an important driver of AMR, and the misuse of antimicrobials is an issue in both sectors, there must be shared effort to reduce human and animal AMU. As one veterinary student explained, “Use in both animals and humans is an issue. If we (vets) are trying to make a difference, we want to make sure human healthcare professionals are trying, too.” Another veterinary student emphasized the importance of collaboration between sectors, mentioning that “Vets won’t be able to address the issue alone. We need to change behaviours of the general public, and that doesn’t include only pet owners. We need to be realistic and know that we can change our own prescribing practices, but overall it’s hard to say what effect that will have without some simultaneous and collaborative initiatives with human health professionals.” Veterinary students also felt that their role in the issue is the same as other health professionals, explaining that “The role of a vet in AMR is on par with other health professionals. You should try to be more stringent with how often you are giving antibiotics, and what kinds of antibiotics you are using, and how long you are prescribing them for. Being sure you are using them for the right thing, and that they are necessary.”

In addition to antimicrobial prescribing practices, both medical and veterinary students felt that physicians and veterinarians can play significant roles in education of patients and the general public, as well as advocacy. Veterinary students highlighted the potential roles of veterinarians, beyond clinical duties and prescribing practices, in depth. As one veterinary student participant explained, veterinarians could be “…involved in public health and dissemination of information—being in the first line of health care along with doctors and nurses and other healthcare professionals. I think that maybe vets aren’t very involved with public health efforts right now, but it could be an interesting new role for them in addition to their usual patient/client interactions.” Another veterinary student participant highlighted the importance of a unified message across all relevant sectors, explaining that “Different health professionals (vets, doctors, nurses) saying the same thing and getting the same message across would have a huge impact on society.” Medical student participants echoed the idea of multi-sectoral collaboration, with one student mentioning “Vets and pharmacists need to be included in the efforts. Microbiologists, basic scientists. Public health needs to be involved too. Governmental representation is needed to speak out on the issue and bring some of these sectors together.”

### Content of communication products about AMR

#### Theme 1: content to address pressure to prescribe antimicrobials

Both medical and veterinary students felt that their future patients and clients needed to know about the issue of AMR, what it is, and how it could impact them, as well as the distinction between viral and bacterial infections. The participants felt that if these aspects were communicated effectively to future patients and clients, it could decrease the pressure to prescribe, and potentially have an impact on AMR. For example, one medical student indicated that “Sometimes it is confusing for patients. Sometimes your infection is caused by bacteria, sometimes it’s caused by a virus. It can send confusing messaging. Half the time we’ll never even know if it was a virus or bacteria if we don’t get to the testing stage. They need a basic understanding of viruses and bacteria and how we would go about treating each.”

Patient pressure to prescribe was described in depth during the focus group with medical students, as were the challenges balancing patient needs and desires with prudent use of antimicrobials. Medical students described their own experiences with demanding patients during school placements or volunteer experience in clinics and discussed how these interactions may influence prescribing behaviours. Students suggested that communication with patients should try and personalize the issue of AMR to highlight its importance, with one student explaining that “If you make it personal, people may be more likely to listen.” Another participant highlighted that when patients have an improved understanding of the issue, they may be less likely to ask for an antimicrobial and the interaction may be less confrontational, explaining that “They need to have a general understanding of AMR—what causes it, why it is important, so that they don’t pressure the physician and they do understand the issue. In that same family clinic I volunteered at, I had a mom come in and her kid had strep throat, and she said “I know if it’s viral you aren’t going to give me anything”. It was nice for the patient to have that understanding and be okay with our treatments. It made the patient encounter super easy.”

Both medical and veterinary students explained that a patient or client’s past lived experiences often influenced their desire for an antimicrobial, as they may have received an antimicrobial for themselves or their pet(s) in the past with successful results, and therefore believe that an antimicrobial prescription is the correct course of action. One medical student explained that “[Patients] need to understand what antibiotics are, rather than assuming that because it made them feel good one time, it’s going to fix all of their problems.” Veterinary student participants reiterated the concern that if a client has given their pet an antimicrobial in the past, they are more likely to want a similar treatment in the future. One veterinary student participant explained that “Many patients with dogs or cats with a simple infection, like a urinary tract infection, will just call the office and ask for an antibiotic prescription instead of bringing their pet in for an examination because their pet has had this happen before and the antibiotic worked.” When asked what veterinary clients should know about AMR, veterinary students felt that they needed an overall understanding of the issue and how complex it is, and they also needed to understand how it could impact their own health, as well as the health of their pets. “You need to make it personal for them, because if they think it’s some big, complex issue, they aren’t going to care. You need posters or handouts or something that explains why you shouldn’t use antimicrobials if you don’t need them, and you need to mention that someday there might not be a drug that can treat your pet’s infection. A lot of people care more about their pet’s health than they do about their own, so you need to mention the human health consequences but mainly focus on how their pet could get very sick without having an effective treatment.”

#### Theme 2: content to improve understanding of AMR among medical and veterinary students

Both medical and veterinary student participants voiced a lack of confidence in their understanding of AMR and expressed a desire to learn more about the issue. Veterinary student participants felt that because AMR is such an important issue and threat to public health, it is imperative to have a thorough understanding of the issue and its underlying drivers. One veterinary student believed they did not know as much as they should about AMR, but acknowledged that they are still early in their veterinary studies, explaining that “I don’t think I have as good of an understanding of the issue as I should. For example, I don’t really know about how the environment plays a role. But I think that as I go on in vet school I will learn more, and I’ll make sure to educate myself, because it’s a really important issue. The implications go beyond the patient and their owner.” Although one medical student participant stated that medical students are “much more educated about it than people in vet school,” this participant also indicated that they don’t have a comprehensive understanding of the issue, explaining that “I don’t know very much about AMR. I just know that I have this kind of general sense that I should avoid prescribing antibiotics whenever possible…”. The lack of understanding was also emphasized by other medical students, with one student mentioning that they “…have no super specific knowledge. But I do have a sense of the importance of preserving the resources that we have right now.” How lack of knowledge and the complexity of AMR affect patient and client interactions was also discussed among both veterinary and medical students. Veterinary students felt that to best communicate the complexity and importance of the issue with their clients, they need to have a comprehensive understanding of the issue themselves. As one veterinary student explained, “It starts with ourselves first. We need to fully understand the issue, what causes it, and how to try and solve the problem before we can expect pet owners to understand. We can’t just be looking at the clinical aspects, because if an animal has a resistant infection, the source of the infection might not be prior antibiotic use in that animal. It could have acquired the infection from the environment, for example. So it’s important for us to understand all of that.” Students also emphasized that they feel it is important that health professionals have a solid understanding of the issue and its drivers in order to educate patients, with one medical student explaining that “…I think that our patients need to know that it’s a complex, broad issue, but I don’t think that physicians necessarily know enough about the non-clinical aspects to communicate these well to their patients. Education needs to start with the physicians before we can be expected to communicate all of these complex things with patients.”

Importantly, both medical and veterinary student participants indicated that communication products targeting students and practicing medical professionals and those targeting patients and clients are of equal importance, and they emphasized the need to consider these different target audiences when developing these products. Students described the need to consider different levels of knowledge of AMR and the importance of tailoring products to the target audience to enhance awareness and information uptake. Furthermore, several participants recognized that one communication product would not be suitable for all target audiences, and a more widespread, comprehensive knowledge translation strategy encompassing many communication products is needed. As indicated by a veterinary student participant, “You need to be aware of differences in knowledge, and what the average person in society is aware of. You need a general, widespread approach to educate and you can’t just use one tool to reach everyone”. A medical student participant echoed these thoughts, suggesting that “There is no one-size-fits-all approach when it comes to communicating this type of information, especially because it is so complex.” The participating students also expressed the need for a broader understanding of the issue and how it will relate to their own practices in the future. Generally, veterinary students felt that it was important for veterinarians to know about the connections between human, environmental, and animal health as they relate to AMR. Veterinary students explained that having a greater understanding of the relationships between these health sectors supported the need for prudent prescribing practices, because the implications of AMR go beyond the patient that they are treating. An increased awareness and understanding of the drivers and impacts of AMR across all sectors can serve as a motivator to “Do things properly, and only prescribe when you need to. You don’t want to be responsible for all of these downstream effects, like making people sick, or contaminating the environment.” In contrast, there was some uncertainty among the medical student participants as to whether this information is useful in practice, though they acknowledged its role in informing advocacy for responsible prescribing. Medical student participants expressed a desire to understand the connections between human, environmental, and animal health, but were unsure of how they would apply it in everyday interactions with their future patients. As one medical student explained, “If you have a patient coming in with a resistant infection, do you think it’s helpful to know about those other things that contribute to the problem? Or do you think it’s better just to be focused on what kind of infection it is, and how to treat it?” Another student felt that it was important that they know about these connections to better understand the potential sources of infection amongst their future patients, but was unsure of whether this information should be communicated to patients, saying “I don’t think that patients having that information would be bad, but I also don’t know how it would impact the interaction with the patient. If a patient comes in with a resistant infection, I don’t think it would change my interaction with that patient, but it might change the way I think about using antibiotics in the future. I would be more likely to advocate for proper antimicrobial use among all sectors, not just human medicine.” Another medical student felt that having information about the complexity of AMR and how the sectors are connected could help them take a more active role in combating the issue in the future, explaining that “…in terms of advocacy, like if I know more about how using antibiotics routinely in prevention and farming, how does that impact human health? If I knew more about that, then I would be able to take a little bit more of a stand in terms of advocating for how we should use these drugs in agriculture and human medicine.”

Across all focus groups, a perceived need for continuing education among practicing physicians and veterinarians was highlighted. Students described their sense of importance for physicians and veterinarians to continue to be informed about developments in the field, suggesting that “…[there are] a lot of practicing physicians who did not start practicing within the last five years, and are not going back to school, so continuing education is important.” This concept was also discussed amongst veterinary student participants, with one student explaining that “Vets need to be continuously updated on new research in the field, especially if it relates to what or how they are prescribing to their patients.”

### Format of communication products about AMR

#### Theme: balancing the quantity of information with visual appeal

The major theme that emerged during discussions of the example communication products was the need to balance the quantity of information with visual appeal of the communication products. When presented with four different communication products, preferences varied, both between the medical and veterinary student focus groups and within each of the respective groups. Overall, students felt that the most useful communication product for themselves as future physicians or veterinarians was the fact sheet, as it contained “A good amount of information so that you are actually learning something, but not too much to overload you to the point where you can’t even determine what the key messages are.” Medical students explained that it could be something they could quickly refer to in practice and extract the important messages because of the length and layout of this type of product. An interesting finding was that both veterinary and medical students found the example infographics to be the least useful communication product. Although they did acknowledge that this format presented information in a digestible way for certain target audiences (e.g., the general public), they felt that it didn’t provide enough information to “…explain why AMR is important, why we should care, and what we should do about it”, and it was therefore challenging to make this type of communication product impactful for this purpose.

The communication product that generated the most conflicting opinions was the example long report, a document produced by the Canadian government to summarize all AMR-related surveillance findings over a period of one year. The participants were asked to focus on the layout and the type of information this report could convey and were asked not to read the report in its entirety or focus on the content of the report. Veterinary students generally disliked this report; although they did acknowledge the report’s utility and ability to convey a large quantity of information, they could not see themselves using this type of KTT material as future practitioners or distributing this to future clients. They also explained that as they transition into practice, they likely would not have time to search through a long report to find the information they needed and would turn to the internet instead. In contrast, the medical student participants felt that this report should always accompany a shorter communication product for those individuals who wanted more information about a specific topic. The medical students felt that they would use this type of communication product regularly in their future practice. Due to the length of the report, they explained that they likely would not use this during a patient interaction, but it would be something they would like to have access to and refer to when needed.

During the veterinary student focus groups, there was a significant amount of discussion surrounding the balance between text information and graphics, with most students preferring a product with more text than visuals. Students felt that more information can be conveyed via text in comparison to graphics, and this would be more useful in their future practice and during interactions with future clients. In terms of communication products for future patients or clients, though, both veterinary and medical student participants felt that graphics are important. Veterinary student participants felt that if a communication product was not visually appealing, it would not attract the target audience and would reduce the uptake of information. Medical student participants echoed these thoughts, indicating that the other communication products may not be as easily embraced as infographics, because there may be too much information that they are not willing to read. Overall, all medical and veterinary students agreed that a comprehensive approach would be needed, and that no single communication product could be used for all target audiences. The participants explained that a variety of communication products, including infographics, fact sheets, and long reports, should be available and distributed to future patients/clients and themselves as practicing physicians and veterinarians in the future. This strategy would ensure that the messages are tailored to the target audience, and members of the target audience can choose to use the products based on the quantity and type of information they desire.

Veterinary students suggested that videos may be an alternative communication product that could be adopted for both professionals and clients, and suggested that “…they could be played on YouTube as ads that you have to watch before you can watch your video. This could be the best way of reaching the younger generations and millennials, because they use YouTube so much already.”

### Dissemination of communication products about AMR

#### Theme: accessibility and reach of communication products

The importance of making communication products accessible to target audiences was discussed in the medical and veterinary student focus groups. Veterinary students felt that distributing communication products to clinics on a regular basis would be effective, particularly for those in small animal practice, rendering products accessible for veterinarians and easily distributed to future clients. The students felt that distributing hard copies of the communication products would be beneficial, as clinics could then post or distribute these as they see fit. The veterinary students acknowledged that some veterinarians outside small animal practice may not be routinely working in a clinic setting and suggested that products also be distributed at industry meetings, conferences, or other events to reach veterinary professionals working across different settings.

Medical students raised similar comments and felt that distributing communication products to clinics would be an effective way of reaching both future practicing physicians and patients. Additionally, students suggested that distributing these products through specialty-specific scientific journals may be the best way of reaching physicians who work in different settings. The medical students explained that many physicians work across multiple clinics or multiple hospitals and distributing products through scientific journals may be the best way to reach individuals with a wide geographic distribution.

Students suggested that social media can play a very important role in disseminating information, as “…it is a really good way to reach the general public. So many people use social media, and this could be a good way to raise more attention and awareness of the issue.” Medical students discussed how reputable sources releasing AMR communication products via social media could have positive impacts, as “…people believe what they read online, so if reputable sources are releasing information, it can maybe combat some of the un-reputable sources, or articles containing false information, that are online.”

## Discussion

This study qualitatively explored both Ontario medical and veterinary student perceptions of AMR to gain an in-depth understanding of the knowledge and attitudes of future prescribers. Overall, the student participants felt that AMR is an important global issue and they generally understood the key drivers of AMR, but still felt they needed additional knowledge and training to better understand how AMR in humans, animals, and the environment is connected. Additionally, participants highlighted the need to consider external influences, such as the media, on perceptions of AMR, as well as a need to develop a variety of communication products tailored to different audiences. Students generally preferred communication products with more text than visuals but acknowledged that visual products (e.g., infographics) may be best for communicating AMR information with the general public.

### Knowledge and perceptions of AMR

Encouragingly, all students generally understood the key drivers and potential impacts of AMR; however, several participants indicated that they had not heard of antimicrobial stewardship, or were unsure if they had heard the term, with fewer medical students reporting recognition of the term compared to veterinary students. This may be attributed to the majority of participants being first year students, as a survey of human and animal health students in the UK found that students in later stages of their respective programs were more likely to have heard of antibiotic stewardship [15]. Another study exploring Australian veterinary students’ knowledge and perceptions of antimicrobial stewardship found that students closer to graduation were more likely to correctly identify the importance of different antimicrobials compared to students who were one year earlier in their veterinary education [20]. This suggests that greater exposure to antimicrobial stewardship principles during the final year of veterinary school may result in improved knowledge of antimicrobial importance ratings. Although there may be differences in curricula between Ontario, the UK, and Australia, it is likely that focus group participants early in their medical or veterinary education may not yet have been exposed to stewardship or its principles. An interesting finding from the focus group discussions was the emphasis on the contribution of AMU in production animals relative to human AMU by the medical student focus group participants. The medical student participants also indicated that they lacked a comprehensive understanding of AMR and its links to the agricultural sector, and had less recognition of antimicrobial stewardship compared to the veterinary student participants. This suggests that enhanced knowledge of AMR, its drivers, and its links across the human and veterinary sectors is needed.

Overall, the medical and veterinary students who participated in this study felt that AMR was an important global challenge. Both the pre- and post-focus group surveys revealed that Canadian students felt that AMR was not as important as the issue of climate change, whereas the UK survey demonstrated that students believed that AMR was a more important global challenge than all other issues, including climate change [15]. The UK study had a sample size of over 200 students, and therefore likely provided a more representative sample compared to our study. This highlights the utility of different research methodologies and approaches; although surveys that are widely disseminated may provide more representative results, focus groups can provide more in-depth understanding of perceptions and views, and context around survey results.

Students felt that the media play important roles in raising awareness around any issue, and they perceived AMR to receive less media coverage compared to other global issues. These findings can be applied to the agenda-setting theory, which describes how the influence of media can affect public perceptions of reports and issues [21]. The relative importance of an issue may determine how often an issue appears in the media, which can influence public perception of importance of the issue [21]. This can also have implications for policies and actions to address the issue, as media coverage and the public agenda can influence the actions and awareness of policy-makers [21].

### Content, format, and dissemination of communication products about AMR

In addition to exploring student perceptions of AMR, this study used focus groups to develop an in-depth understanding of how future physicians and veterinarians believe we should communicate information about AMR to them and their future patients and clients. A theme that emerged in both the veterinary and medical student focus group discussions was the influence of patients or clients on prescribing practices, and students highlighted that the content of future AMR communication products should address this challenge. Medical students raised the idea of future patients pressuring physicians to prescribe an antimicrobial and highlighted how it was sometimes easier to give into this pressure, rather than attempting to influence patient knowledge and attitudes on the issue of AMR. Similarly, veterinary students discussed the impact of pet owners on prescribing practices; some pet owners may pressure veterinarians to prescribe an antimicrobial to their pets due to an unwillingness or inability to pay for diagnostic testing. The issue of patient pressure to prescribe an antimicrobial has been well documented in the literature, with studies showing that patient pressure, or the perceived expectation of the patient by physicians to receive an antimicrobial, is associated with antimicrobial prescribing decisions [22, 23]. Perceived pressure from clients to prescribe an antimicrobial has also been identified as a barrier to appropriate antimicrobial prescribing in veterinary medicine [24, 25]. Overall, students felt that pressure to prescribe may be reduced if their future patients and clients had a better understanding of the issue of AMR, but they voiced a lack of confidence in their own knowledge of the complexity of AMR and its drivers, and therefore lacked confidence in their ability to educate these future patients and clients on the issue.

The student participants suggested that communication products about AMR aimed at future patients and clients should attempt to personalize the issue to improve understanding of its overall importance and implications. Personalizing the issue may facilitate AMR-related behavioural change, or changes in perception of the issue. This could be done using a theoretical framework such as the Health Belief Model (HBM), which is used to examine the uptake of behaviours, and is a guiding framework for health-related behavioural interventions [26]. The idea of creating communication products that are personal to the target audience ties into the perceived susceptibility construct of the HBM, which states that individuals must believe that they are at risk of acquiring an illness or disease in order to change health-related behaviours [26, 27]. Risk perception must be considered when developing and identifying key messages for future AMR communication products aimed at the general public. If patients and veterinary clients understand that AMR can negatively impact themselves, their pets, and those around them, they may be less likely to pressure their healthcare professional to prescribe an antimicrobial for themselves or their pets. A survey administered to individuals seeking care at a medical clinic found that communication strategies focusing on harm to the individual or those close to them had the most impact on patient likelihood to request antibiotics [28]. Future communication products should frame the issue of AMR in a way that is relevant to the target audience (e.g., patients, pet owners, food animal producers) by using examples and explaining the potential of resistant infections to affect anyone, including both humans and pets [29]. Although statistics provide valuable information on the importance and the magnitude of a public health issue, using stories involving real people affected by AMR may better resonate with the public than facts and figures, making the potential impacts of AMR more relatable and enhancing support for behavioural change [29].

The format and method of delivery of information was also discussed with the focus group participants, with the participants highlighting the need for different communication formats for future physicians, veterinarians, and their future clients/patients. Communication products for future medical patients and veterinary clients are needed to enhance understanding and awareness of the issue of AMR, but the students also felt that they themselves need more information to better explain the content of communication products if their patients and clients have questions. The medical and veterinary students felt that communication products with more visuals, such as infographics and videos, could be helpful for members of the general public (i.e., future patients or clients), but preferred fact sheets, with more detailed information, for communicating AMR information to themselves as future health practitioners. Public health agencies and other AMR stakeholders must consider the specific needs of their target audiences to develop effective AMR communication products. There is no “one size fits all” approach to communicating AMR information, as there is considerable variation in the key messages and level of detail needed by different stakeholders, including healthcare professionals, pet or livestock owners, and the general public. A comprehensive communication strategy should carefully consider the different target audiences that need to be reached, the key messages that need to be communicated consistently about AMR, and the most effective type of communication product for each audience.

Furthermore, the potential role of social media in disseminating communication products was discussed several times across the veterinary student focus groups, as well as in the medical student focus groups. Student participants felt that social media is a tool that can be used to reach specific target audiences, particularly those with younger demographics, as the participants felt that these age groups are more likely to engage in social media. Previous studies have demonstrated that the Internet and social media in particular are commonly used for seeking information related to antibiotics [30, 31]. Additionally, an analysis of Twitter activity over a six-month period was conducted to determine how this platform was being used as a tool for antimicrobial stewardship, and identify the most influential users who focus their Twitter activity on stewardship [32]. Physicians, medical journals, pharmacists, and health institutions were identified as key influencers or opinion leaders on Twitter in the area of antibiotics, highlighting the potential for social media to communicate messages related to antimicrobials and the important of stewardship. Another analysis of Twitter-use related to both AMR and antibiotics identified health news sources, and others working within the medical and pharmaceutical fields as influential users that facilitate conversations about AMR and antibiotics on this platform [33]. These results highlight the need to develop comprehensive knowledge translation strategies that incorporate social media to disseminate key messages about AMU and AMR, particularly by individuals who are viewed as leaders in the field. A systematic review of the effectiveness of AMR interventions amongst the general public found that the use of social media in interventions designed to improve awareness of AMR was lacking, despite the increasing popularity of social media in society [34]. As people continue to increasingly seek out health information online, social networking sites are important places for dialogue and enhancing awareness and understanding of complex issues. Some communication products targeted at the general public, such as infographics, are easily sharable through social media channels, further enhancing the appeal of incorporating social media into communication strategies [35]. Additionally, key influencers can engage in dialogue with other stakeholders in the AMR issue (e.g., other health professionals) to share research findings, and messages regarding antimicrobial stewardship.

### Considerations and limitations

This study provided an in-depth exploration of medical and veterinary student perceptions of AMR and preferences for AMR knowledge translation and communication strategies; however, there are several limitations to this research. Firstly, participant recruitment for the focus groups was challenging, and resulted in fewer focus groups being conducted than originally planned. Several institutions had ethics processes in place for conducting research with students, and these processes often required a local investigator to oversee the study. Because our research team was largely based at a single institution, it was difficult to access the student populations at other institutions. Additionally, few responses to the recruitment email and scheduling issues with interested students were also challenges when recruiting medical students at several universities. In the future, it would be interesting to distribute the survey used in the focus group discussions to medical and veterinary students across Canada to obtain a larger sample size and more representative results.

Secondly, there is likely to be some bias arising from the focus group discussions, particularly with the medical student focus group. Overall, there was a lack of diversity in the sample of medical and veterinary students that participated in the discussions in terms of year of study. The focus groups conducted with veterinary students did have at least one participant from every year of veterinary school, but the majority of students were in their first year of study. Additionally, as a result of the recruitment challenges outlined previously, only one focus group was conducted with medical students, and most of the participants in this focus group were first year students. A more equal distribution across the four years of medical and veterinary school may have provided additional perspectives on the issue of AMR, and future studies could target students that are farther along in their studies, as they may have different perspectives as a result of completing more coursework and potentially having more clinical experience.

It is also important to acknowledge that this study explored student perceptions about the issue of AMR, and their perceptions of the content, format, and dissemination strategies that are needed to communicate information about AMR to themselves as future medical professionals, as well as their future clients. The participants in the focus groups were medical and veterinary students, and not healthcare professionals that are currently working in the field. Healthcare professionals may have different views on AMR, and different communication preferences compared to students training to be professionals based on their experiences with different cases, patients and/or clients. Additionally, this study captures the students’ current views on the issue of AMR in their early years of study, and it is impossible for them to predict their knowledge of AMR upon graduation. As these students complete their education and move into the realities of day-to-day practice, their views on the issue and preferred methods of communication may evolve. However, it is important to explore the perceptions and communication preferences of students, as they represent the next generation of practitioners and antimicrobial prescribers. These perceptions and preferences need to be validated with actual medical practitioners in the field to determine how practitioners would want to receive information about AMR. Future research could seek to repeat this study with actual medical and veterinary health practitioners, and compare and contrast the perceptions related to AMR and communication between these practitioners and the medical and veterinary students.

## Conclusion

In conclusion, this research demonstrated that Ontario medical and veterinary students recognize the importance of AMR as a public health issue, and the reliance of human and veterinary medicine on effective antimicrobials. By investigating medical and veterinary student perspectives, public health agencies and researchers will be better positioned to develop effective, targeted communication tools to address AMR in each of these groups of future health professionals. Future research should attempt to incorporate other health professionals, such as pharmacists, nurse practitioners, and dentists. These groups can dispense and/or prescribe antimicrobials to patients, and they play important roles in primary health care in many communities. Continuing this research and incorporating multiple, diverse viewpoints from future and current antimicrobial prescribers will help further elucidate the key messages, format, and method of delivery for communication tools aiming to address this significant public health issue.

## Electronic supplementary material

Below is the link to the electronic supplementary material.

Supplementary Material 1

Supplementary Material 2

Supplementary Material 3

Supplementary Material 4

## Data Availability

The datasets used and/or analyzed during the current study are available from the corresponding author upon request.

## List of abbreviations

AMR: antimicrobial resistance
AMU: antimicrobial use
