# Supplementary material for: Exploring medical and veterinary student perceptions and communication preferences related to antimicrobial resistance in Ontario, Canada using qualitative methods
Source: BMC Public Health. 2023 Mar 13;23:483. doi: 10.1186/s12889-023-15193-x (PMC10012462; doi:10.1186/s12889-023-15193-x)
Supplement: Supplementary file 1 — Supplementary Material 1 [file 12889_2023_15193_MOESM1_ESM.docx]

**Additional file 1 (.docx)**

- **The semi-standardized focus group guide used during the discussions**

**Focus Group Guide**

***Introduction to Participants and AMR***

1. Tell us who you are (first name), what year of the M.D., PharmD, or DVM program you are in, and what you most enjoy doing outside of medical/veterinary school.
2. Tell me what you know about antimicrobial resistance (AMR).
3. In the future, you will be practicing physicians/veterinarians/pharmacists. What is the role of a physician/veterinarian/pharmacist in the issue of AMR?

Basic introductory slides will be presented to the group to establish a baseline understanding of how the student researcher is describing AMR.

***Content of Future Communication Materials***

1. What do physicians/veterinarians/pharmacists need to know about AMR, outside of the clinical realm?

- Is it important for physicians/veterinarians/pharmacists to know about the social, cultural, and economic drivers of AMR? Why?
- Is it important for physicians/veterinarians/pharmacists to know about the environmental and foodborne aspects of AMR? Why?
- Is it important for physicians/veterinarians/pharmacists to know about the links between human, environmental, and animal health, and the drivers of each of these aspects?

1. What do your patients/clients need to know about AMR?

- For example, what do they need to know about the social and economic drivers of the issue?
- Is it important for patients to know about the links between human, environmental, and animal health? Why?

***Format of Future Communication Materials***

The group will be presented with communication materials, such as an infographic, a short summary, a fact sheet, and a long report relating to AMR.

**Note that the focus of the participants should be on the format of each communication piece, rather than their content.*

1. For each communication piece:
   1. What do you think about this type of communication material?
   2. What are the strengths of using this type of material to communicate AMR information (overall, not relating to the content on it)?
   3. What are the weaknesses of using this type of material to communicate AMR information?
2. You were presented with different options for AMR communication materials. What is the most effective method of communicating the complexities of AMR to you in the future, as someone diagnosing and prescribing antimicrobials?

- Which format did you prefer? Which was the most useful?
- How could you use these in the future?
- What would be the most effective method of communicating the relevant information to your clients/patients?

1. What other formats, if any, would be effective at communicating this information?

- Would videos be used to communicate this information? Webinars?
- Could social media play a role in communicating this information?

***Transmission/Delivery of Future Communication Materials***

1. Once you are practicing physicians/veterinarians/pharmacists, how would you like to receive information about AMR?

- What type of information would you like to receive?
- What is the best way to deliver this information? (e.g. mailing lists, etc.)
- What would be the best way to access this information in practice?
