# Supplementary figures and images for: Exploring medical and veterinary student perceptions and communication preferences related to antimicrobial resistance in Ontario, Canada using qualitative methods
Source: BMC Public Health. 2023 Mar 13;23:483. doi: 10.1186/s12889-023-15193-x (PMC10012462; doi:10.1186/s12889-023-15193-x)

**Additional file 2 (.docx)**

- **The slides presented during the focus group discussions**


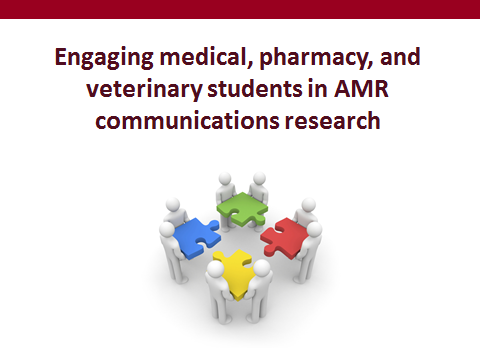


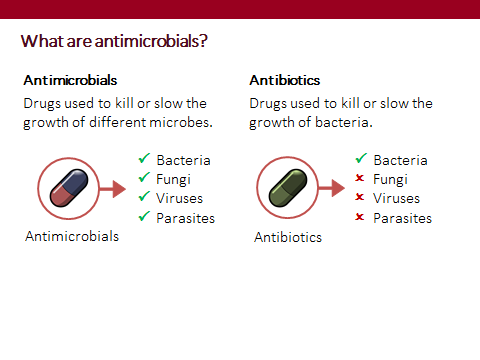


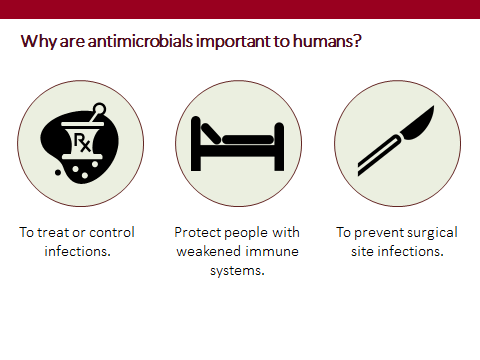


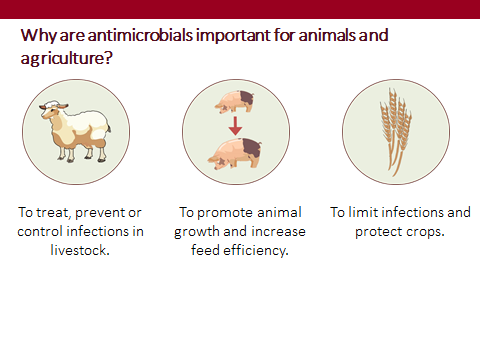


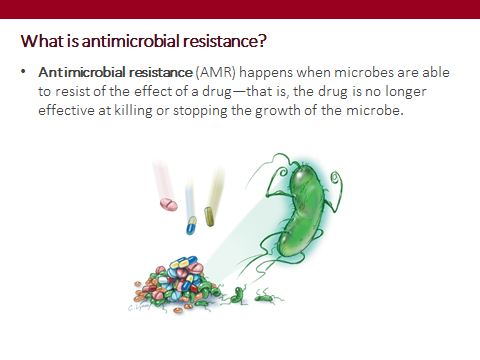


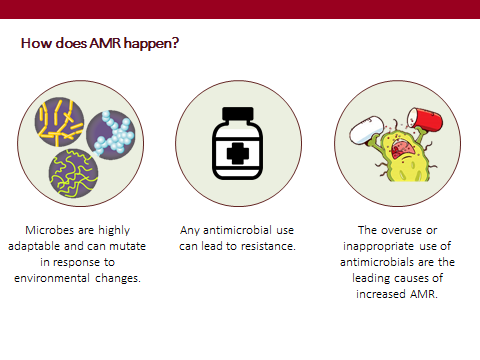


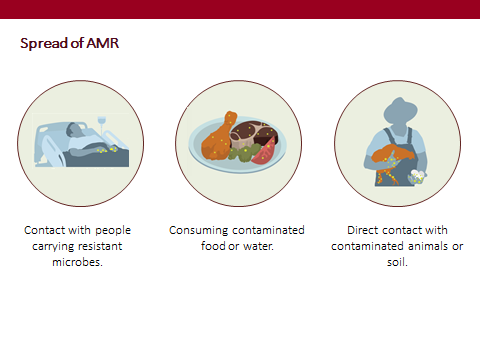


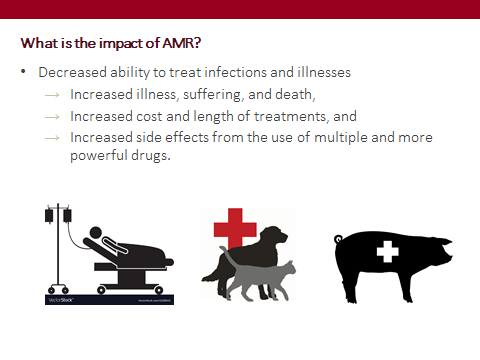


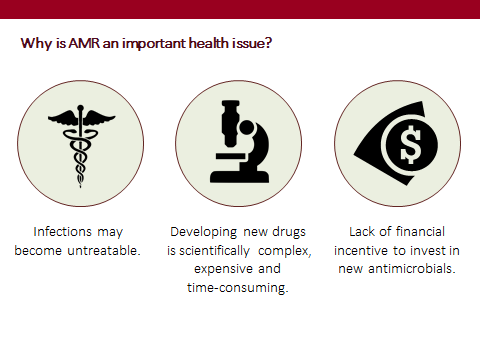


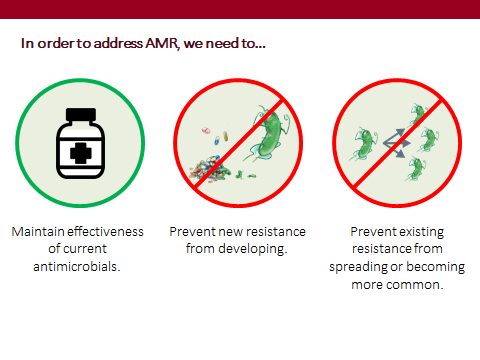

Supplement: Supplementary file 2 — Supplementary Material 2 [file 12889_2023_15193_MOESM2_ESM.docx]
