# Supplementary material for: Exploring medical and veterinary student perceptions and communication preferences related to antimicrobial resistance in Ontario, Canada using qualitative methods
Source: BMC Public Health. 2023 Mar 13;23:483. doi: 10.1186/s12889-023-15193-x (PMC10012462; doi:10.1186/s12889-023-15193-x)
Supplement: Supplementary file 3 — Supplementary Material 3 [file 12889_2023_15193_MOESM3_ESM.docx]

**Additional file 3 (.docx)**

- **The survey administered to focus group participants**

**Focus Group Participant Survey**

1. **Please rank the following global issues in order of importance on a scale of 1 to 5 (1 = not important, 5 = extremely important].**

_______ Climate Change

_______ Antimicrobial Resistance

_______ Terrorism

_______ Food Security

_______ Chronic Diseases

Prefer not to answer

1. **Antimicrobial resistance can impact which of the following (select all that apply):**

The environment

Human health

Animal Health

Unsure

Prefer not to answer

1. **In your opinion, which of the following contributes to the issue of antimicrobial resistance? Select all that apply.**

Antimicrobial use in hospitals

Antimicrobial use in the environment

Antimicrobial use in the community

Antimicrobial use in companion animals (e.g. dogs, cats, etc.)

Antimicrobial use in food-producing animals (e.g. cattle, pigs, chicken, etc.)

Other (please specify) ___________________________________________________________

Prefer not to answer

**Have you ever heard of the term “antibiotic stewardship” or “antimicrobial stewardship”?**

Yes

No

Unsure

Prefer not to answer
