# Supplementary material for: Exploring medical and veterinary student perceptions and communication preferences related to antimicrobial resistance in Ontario, Canada using qualitative methods
Source: BMC Public Health. 2023 Mar 13;23:483. doi: 10.1186/s12889-023-15193-x (PMC10012462; doi:10.1186/s12889-023-15193-x)
Supplement: Supplementary file 4 — Supplementary Material 4 [file 12889_2023_15193_MOESM4_ESM.pdf]

Additional file 4 (.pdf)

- **Examples of the communication materials presented to participants**

Example of infographic #1

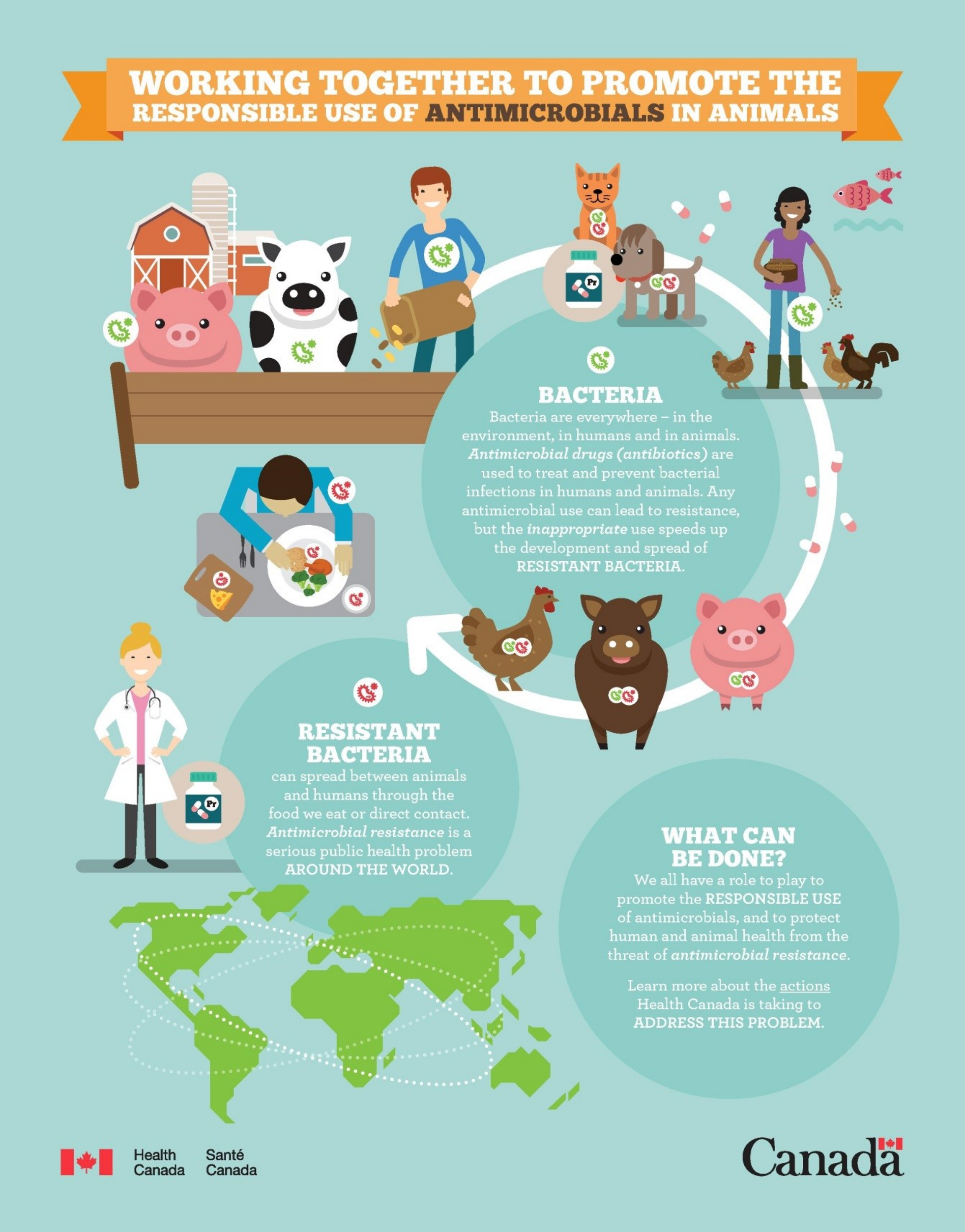

Example of infographic

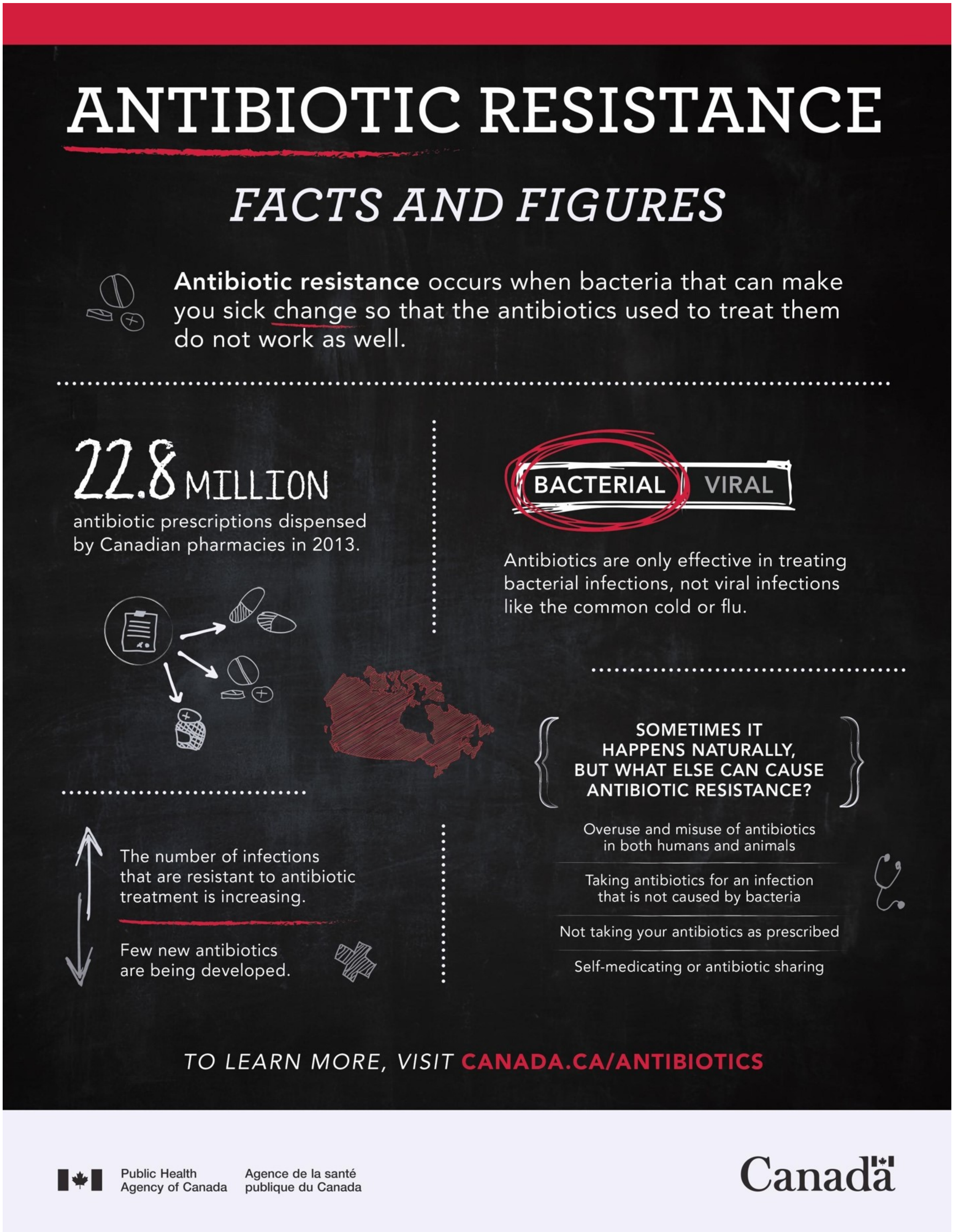

Example of fact sheet

Public Health Agency of Canada | Agence de la santé publique du Canada

PRESCRIBE ANTIBIOTICS WISELY

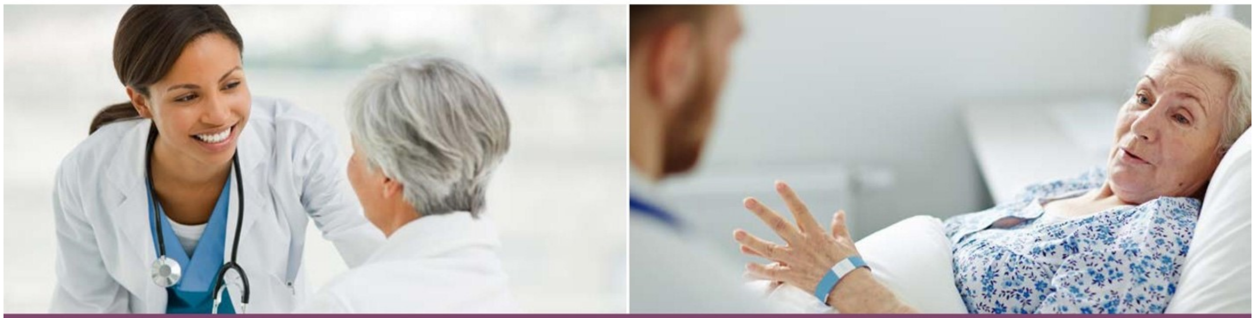

The overuse and misuse of antibiotics is contributing to the development of antibiotic resistance.

Adults aged 60 years and older are prescribed antibiotics 1.5 times more than any other age group in Canada.

Asymptomatic bacteriuria and **upper respiratory infections**, including sinusitis, are the most likely conditions where antibiotics are prescribed unnecessarily.

- Up to 80% of patients with asymptomatic bacteriuria receive antibiotics. This can lead to adverse events such as *C. difficile* infections.
- Antibiotics are rarely indicated for upper respiratory illnesses, which are often viral or, if bacterial, are typically self-limited.

KEY CLINICAL POINTS

Asymptomatic bacteriuria:

- The presence of bacteria in the bladder/urine without symptoms pertaining to the urinary tract is referred to as asymptomatic bacteriuria. This represents a colonization state rather than a bacterial infection.
- Pyuria (white blood cells in urine) is very common in asymptomatic bacteriuria and is not an indication for antibiotic therapy.
- Screening urine for bacteriuria or pyuria in an asymptomatic patient or a patient with non-specific symptoms is not recommended.

Canada

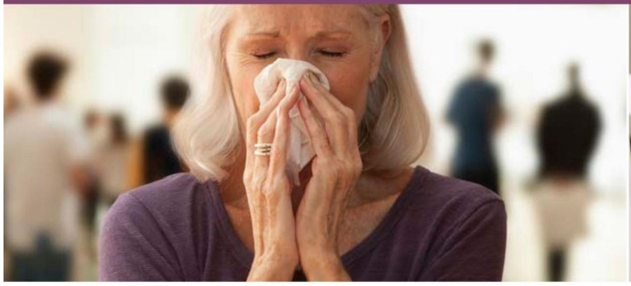

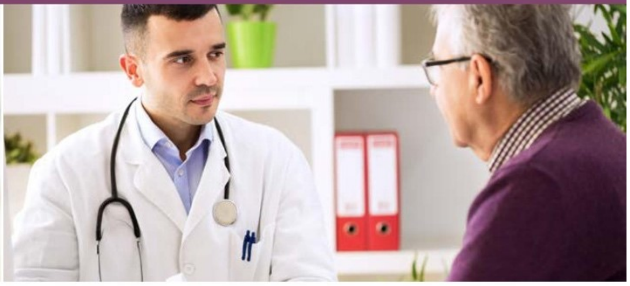

› Foul smelling, cloudy urine typically indicates dehydration and not a urinary tract infection. These alone are not an indication for sending a urine sample for analysis.

› Non-catheterized patients will typically have specific or localizing symptoms to the urinary tract, such as dysuria, frequency or urgency when a urinary tract infection is present (in older women, acute dysuria is the most discriminating symptom for a urinary tract infection).

› Do not attribute delirium to a urinary tract infection before considering:
  - Dehydration
  - New medication/drug interaction
  - Sleep disturbances
  - Trauma
  - Hypoxia
  - Hypoglycemia
  - Infections other than urinary tract infection

Upper respiratory infections:

› Do not prescribe antibiotics in older adults for:
  - Bronchitis
  - Uncomplicated sore throats (note that strep throat is uncommon in older adults)
  - Uncomplicated otitis media
  - Upper respiratory infections, including influenza-like illness as these tend to be viral in origin
  - Sinus infections of less than seven days in duration
  - Cough, unless bacterial pneumonia is suspected. The mean duration of a viral cough is 18 days. Note that tachypnea is an important sign that may indicate pneumonia.
  - Acute asthma exacerbations without clear signs of bacterial infection

Vaccinations:

› As a preventative measure, review current vaccinations for older patients and ensure they are up to date.

For more information, visit CANADA.CA/ANTIBIOTICS

REFERENCES:

- British Columbia. Provincial Academic Detailing Service. *Understanding Asymptomatic Bacteriuria*. Prepared by Dr. Edith Blondel-Hill (MD, FRCP, Medical Microbiologist/Infectious Disease Specialist, Medical Director of the *Do Bugs Need Drugs?* Program), April 2016.
- Choosing Wisely Canada. Antibiotic Wisely campaign.
- Canada. Public Health Agency of Canada. Canadian Antimicrobial Resistance Surveillance System (CARSS) 2017 Report, 2017.

© Her Majesty the Queen in Right of Canada, as represented by the Minister of Health, 2018

Cat.: H14-256/2018E-PDF | ISBN: 978-0-660-25627-6 | Pub.: 170525

Example of short summary

CANADIAN ANTIMICROBIAL RESISTANCE ALLIANCE CARA

ANTIBIOTIC-RESISTANT BACTERIA IN CANADA

New data have demonstrated that MRSA and other virulent organisms are much more prevalent in Canadian hospitals than previously believed. Multi-drug resistant pathogens are invading hospitals as well as the community throughout Canada. These include: methicillin-resistant Staphylococcus aureus (MRSA), vancomycin-resistant enterococci (VRE) and extended-spectrum beta lactamases (ESBL).

MRSA IN CANADA

- *Staphylococcus aureus (S. aureus)* is an especially prevalent bacterium due to its surface proteins, which allow the organism to bind to tissues and foreign bodies coated with collagen, fibronectin, and fibrinogen. This permits the bacteria to adhere to devices such as sutures, catheters, and prosthetic valves. Other medically important staphylococci include *S. epidermidis* and *S. saprophyticus.*

- At any time, multiplying *S. aureus* can overwhelm local defense mechanisms and invade the lymphatic system or the bloodstream – a serious complication that allows the bacteria to invade other tissues including the heart (endocarditis), lungs (pneumonia) or bone (osteomyelitis).

- Methicillin-resistant *S. aureus* (MRSA) is the term used to describe organisms that are resistant to commonly used antibiotics. Methicillin was an antibiotic used many years ago to treat patients with *Staphylococcus aureus* infections. It is now no longer used except as a means of identifying this particular type of antibiotic resistance.

- Another trend recently identified is the rise of community-acquired MRSA (CAMRSA) which has been seen among athletes, soldiers, prisoners, aboriginals and IV drug users.

VRE IN CANADA

- Enterococcus is a bacterium that can be found in the intestines of healthy mammals such as humans and farm animals. However, this gram-positive bacterium can also lead to serious illness and is commonly identified as one of the prime sources of nosocomial (or hospital-acquired) infections.

- Vancomycin-Resistant Enterococcus (VRE) is a mutant strain of Enterococcus that originally developed in individuals who were exposed to the antibiotic vancomycin. It was first identified in France in the late 1980's.

- Vancomycin is a powerful antibiotic that is often used as a last resort. It is generally limited to use against bacteria that are already resistant to other antibiotics.
- VRE infections are especially aggressive and have been associated with mortality rates approaching 60-70%.

ESBLS IN CANADA

- Extended-spectrum beta-lactamases (ESBLs) are a group of Gram-negative bacteria, such as *E.coli* and *Klebsiella* species. ESBLs produce enzymes that break down antibiotics, rendering them useless. Traditional first-line treatment drugs (eg. cephalosporins) are no longer effective if the infection is caused by ESBL-producing bacteria. There are few Canadian studies of this growing category of resistant bacteria, but it is of increasing concern to microbiologists and infectious diseases specialists.
- ESBLs are passed from person to person directly or indirectly, via faecal contamination of hands and objects and then introduced to patients. Currently this is being found most often in hospitals and long term care setting. The problem is usually first identified, however, when an individual presents to hospital and a specimen of urine, blood or wound is submitted to the microbiology lab for culture. (Kingston General Hospital, Infection control – reference)
- The first hospital outbreak of an ESBL-containing (SHV-2) Gram-negative organism was reported in Germany in 1983. (Knothe H, Shah P, Krcmery V, Antal M, Mitsuhashi S. Transferable resistance to cefotaxime, cefoxitin, cefamandole and cefuroxime in clinical isolates of *Klebsiella pneumoniae* and *Serratia marcescens*. Infection 1983; 11:315-7.)

The example of the long report can be found at the following link:
https://www.canada.ca/content/dam/phac-aspc/documents/services/publications/drugs-health-products/antibiotic-resistance-antibiotique/antibiotic-resistance-antibiotique-2016-eng.pdf
